# Development of a Multivariate Predictive Dissolution Model for Tablets Coated with Cellulose Ester Blends

**DOI:** 10.3390/ph13100311

**Published:** 2020-10-15

**Authors:** Eman M. Mohamed, Tahir Khuroo, Hamideh Afrooz, Sathish Dharani, Khaldia Sediri, Phillip Cook, Rajendran Arunagiri, Mansoor A. Khan, Ziyaur Rahman

**Affiliations:** 1 Irma Lerma Rangel College of Pharmacy, Texas A&M Health Science Center, Texas A&M University, College Station, TX 77843, USA; eman_nabil24@tamu.edu (E.M.M.); Khuroo@tamu.edu (T.K.); baran.afrooz@gmail.com (H.A.); dharani@tamu.edu (S.D.); khaldia.sediri@tamu.edu (K.S.); mkhan@tamu.edu (M.A.K.); 2 Department of Pharmaceutics, Faculty of Pharmacy, Beni-Suef University, Beni-Suef 62514, Egypt; 3 Laboratory of Applied Chemistry, ACTR Univ. Ain Temouchent DGRCT, BP 248, 46000 Ain Temouchent, Algeria; 4 Eastman Chemical Company, Kingsport, TN 37662, USA; pcook@eastman.com (P.C.); rajendranarunagiri@eastman.com (R.A.)

**Keywords:** cellulose acetate butyrate, cellaburate, diclofenac, dissolution, hyperspectroscopy, multivariate

## Abstract

The focus of the present investigation was to develop a predictive dissolution model for tablets coated with blends of cellulose acetate butyrate (CAB) 171-15 and cellulose acetate phthalate (C-A-P) using the design of experiment and chemometric approaches. Diclofenac sodium was used as a model drug. Coating weight gain (X_1_, 5, 7.5 and 10%) and CAB 171-15 percentage (X_2_, 33.3, 50 and 66.7%) in the coating composition relative to C-A-P and were selected as independent variables by full factorial experimental design. The responses monitored were dissolution at 1 (Y_1_), 8 (Y_2_), and 24 (Y_3_) h. Statistically significant (*p* < 0.05) effects of X_1_ on Y_1_ and X_2_ on Y_1_, Y_2,_ and Y_3_ were observed. The models showed a good correlation between actual and predicted values as indicated by the correlation coefficients of 0.964, 0.914, and 0.932 for Y_1_, Y_2,_ and Y_3_, respectively. For the chemometric model development, the near infrared spectra of the coated tablets were collected, and partial least square regression (PLSR) was performed. PLSR also showed a good correlation between actual and model predicted values as indicated by correlation coefficients of 0.916, 0.964, and 0.974 for Y_1_, Y_2_, and Y_3_, respectively. Y_1_, Y_2,_ and Y_3_ predicted values of the independent sample by both approaches were close to the actual values. In conclusion, it is possible to predict the dissolution of tablets coated with blends of cellulose esters by both approaches.

## 1. Introduction

Dissolution is one of the quality control tests to measure the performance of a drug product and is required by regulatory agencies [1,2]. Even though it does not completely simulate in vivo conditions, it can be used as a surrogate of the in vivo behavior of certain drugs [3,4,5]. Dissolution determines whether dosage forms is an immediate or extended release formulations, which will eventually determine the duration of the in vivo action and hence the frequency of administration. The rate of drug dissolution can be modulated by the design of solid oral dosage forms, which are broadly classified into monolithic matrix and encapsulated systems [6]. In a monolithic matrix, hydrophilic and/or hydrophobic polymers/excipients control the drug release. The swelling and solubility characteristics of hydrophilic polymer/excipients determine the dissolution of drugs [7,8], while the dissolution mechanism is erosion in a hydrophobic polymer/excipients-based matrix system [9]. In an encapsulated system, the functional coating of the polymer acts as a rate controlling membrane that controls the diffusion of drug from the dosage forms. The encapsulated system can be single (coated tablets) or multiple units (beads or multiparticulate) [10,11]. The dissolution in coated tablets is controlled by properties of the film such as composition, thickness, pore size, number of pores, coating weight, etc. [12,13]. Typically, dissolution is empirically determined, which is a tedious and time-consuming process. Predictive dissolution models based on the empirical data for dosage forms can be developed. It is widely reported in the literature for various dosage forms [14,15]. This methodology hastens the formulation development and predicts the dissolution behavior with future changes in the formulation or coating variables.

Cellulose acetate butyrate (CAB) is a mixed cellulose ester that contains acetate and butyrate esters. The manufacture of CAB involves the esterification of cellulose with acetic anhydride and butyric anhydride in the presence of a sulfuric acid catalyst [16]. The polymer is widely used as a coating material in various industries e.g., aerospace, automobile, industrial electronics, wood, etc. It is also used as an excipient in the pharmaceutical industry. In the FDA ‘Inactive Ingredient Database’ and the United States Pharmacopeia, CAB is listed as ‘Cellaburate’ [17,18]. The literature has reported its application in fabrication of various drug delivery systems, e.g., nanoparticles [19], microspheres [20,21], colonic system [22,23], matrix tablets [24], gastroretentive [25], osmotic [26], and so on. It can also be used as a coating polymer for sustained/extended release of drugs. We demonstrated utility of CAB as a coating polymer for extended release characteristics when used alone or delayed with extended release characteristics when combined with an enteric polymer [27]. The enteric polymer in the polymer blends dissolved at alkaline pH leaving behind pores for water infusion and drug diffusion. The drug release is controlled by the polymer blend composition and coating percentage applied to the tablets beside the physicochemical properties of the drug and processing factors [28,29]. It is desirable to predict the dissolution of the drugs from the coated tablets with the blend of polymers. This can be done using statistical methods called design of experiment and multivariate analysis [30,31]. The objective of the present work was to develop predictive dissolution models of tablets coated with blends of CAB and cellulose acetate phthalate (C-A-P) polymer using experimental design and chemometric approaches and characterize the film by non-destructive methods. Near infrared spectroscopy data was used for the chemometric dissolution model development. Diclofenac sodium was used as a model drug for coating the core tablets with the polymer blends.

## 2. Materials and Methods

### 2.1. Materials

CAB 171-15 and C-A-P polymers were obtained from Eastman Chemical Company, Kingsport, TN, USA. DFS were purchased from Leap Chem, Hangzhou, China. Acetone, acetonitrile (ACN), monobasic potassium phosphate, croscarmellose sodium (CCS), magnesium stearate (MGS), polyethylene glycol 400 (PEG, Fisher Scientific, Asheville, NC, USA), hydroxypropyl cellulose (HPC, MW 100,000, Sigma-Aldrich, St Louis, MO, USA), lactose monohydrate (LMH, Supertab 145D, Mutchler Inc, Harrington Park, NJ, USA), and microcrystalline cellulose (MCC, Vivapur^®^ 102, JRS Pharma, Patterson, NY, USA) were used as received. In-house water (18 MΩ.cm, Millipore Milli-Q Gradient A-10 water purification system) was used in the study.

### 2.2. Methods

#### 2.2.1. Preparation of Core Tablets

The composition of the core tablets consisted of 25% DFS, 47.25% LMH, 14% MCC, 1.25% HPC, 10% CCS, and 2.5% MGS. All the components except MGS and CCS were sieved through a #18 screen, blended (V-blender, Model VH-2), granulated with water (25% *w*/*w* of powder) in a high shear granulator (KG5, KEY International Inc., East Windsor, NJ, USA), dried at 50 °C (Binder In, Bohemia USA) to a target loss on drying of ≤2% *w*/*w* and milled (*Quadro Comil*^®^, Model-193, Quadro Engineering Inc., Waterloo, ON, Canada). The milled granules were sieved through a #18 screen followed by the addition of CCS and lubrication with MGS for 2 min in a V-blender. The final blend was compressed into tablets using Mini Press-1 (Globe Pharma, New Brunswick, NJ, USA) 10-station tableting machine with 8 mm biconvex punches (Natoli Engineering Company, Saint Charles, MO, USA). The core tablets were characterized for friability (USP friability tester, Varian Inc., Cary, NC, USA), hardness (VK 200, Varian Inc., Cary, NC, USA), disintegration (USP disintegration tester in 900 mL water at 37 °C, VK 100, Agilent Technologies, Santa Clara, CA, USA), and dissolution.

#### 2.2.2. Coating Process

Full factorial design was used to build the predictive dissolution model. Independent variables selected were coating weight (X_1_) and proportion of CAB 171-15 relative to C-A-P in the blend (X_2_). Both independent variables were selected at three level as per Table 1. The core tablets were coated in 8” Vector Hi-Coater (Model HCT Mini, Freund Vector, Marion, IA, USA). The coating formulations consisted of 9.6% *w*/*v* (1.6% *w*/*v* PEG and 8.0% *w*/*v* polymer blends) solution in acetone. The components present in the coating formulation were CAB 171-15, C-A-P, and PEG. The PEG and polymer blend proportion was 16.7 and 83.3%. Only CAB 171-15 and C-A-P proportion varied as per Table 1 while PEG was kept constant in all coating compositions. Approximately 400 gm of core tablets were transferred into the pan. The tablets were prewarmed for 15 min at 80–90 °C before coating. The coating process parameters were: Inlet temperature 80–90 °C; core tablets bed temperature 55–70 °C; exhaust temperature 35–40 °C; pan rotation speed 40 rpm; atomization pressure 1.5–2.0 bar; spray rate 8 gm/min; and tablet bed to spray gun distance 10 cm. The coating of the tablets was monitored by measuring weight gain. The coated tablets were dried for 15 min at 70 °C and were characterized for disintegration (USP disintegration tester in 900 mL 0.1 N HCl for 2 h at 37 °C, VK 100, Agilent Technologies, Santa Clara, CA, USA) and dissolution (0.1 N HCl and 0.2 M phosphate buffer pH 6.8, Model 708-DS with 850-DS autosampler, Agilent Technologies, Santa Clara, CA, USA).

#### 2.2.3. Scanning Electron Microscopy

The surface morphology of the coated tablets was studied by scanning electron microscopy (SEM, JSM-7500F, JEOL, Tokyo, Japan). The tablets were approximately 8 nm coated with carbon using sputter coater (Cressington, 208 HR with MTM-20 High-Resolution Thickness Controller) under high vacuum (argon gas pressure 0.01 mbar) and a high voltage of 40 mV. The morphology was captured at a working distance of 15 mm, an accelerated voltage of 5 KV, and an emission current of 20 μA.

#### 2.2.4. Near Infrared Hyperspectroscopy

Via-Spec II Hyperspectral Imaging system was used to collect NIR hyperspectral images of the coated tablets. The instrument details and method of analysis were described in our previous publications [32,33,34]. The data acquisition software used was from Middleton Spectral Vision (Middleton Spectral Vision, Middleton, WI, USA) and data analysis software was from Prediktera EvinceTM (Prediktera AB, Umea, Sweden).

#### 2.2.5. Near Infrared Spectroscopy

The FTIR spectra of coated tablets were collected using a modular NicoletTM iSTM 50 (Thermo Fisher Scientific, Austin, TX, USA). After the instrument passed the diagnostic tests and reflectance standardization, the tablet was placed on the sample window and centered with an iris. NIR spectra ranging from 4000 to 10,000 cm^−1^ with a data resolution of 4 cm^−1^ and 32 scans were obtained in 10 replicates from both sides of the tablets. Omnic v9 software (Thermo Fisher Scientific, Austin, TX, USA) was used to collect and analyze the data.

#### 2.2.6. HPLC Method

The published HPLC method was modified and validated for dissolution sample analysis [27,35]. The HPLC consisted of Agilent 1260 series (Agilent Technologies, Wilmington, DE, USA) equipped with a quaternary pump, online degasser, column heater, autosampler, and UV/Vis detector. The separation of the analyte was achieved on a 4.6 × 150 mm, 5 µm Luna C18 (Phenomenex, Torrance, CA, USA) column and a C18, 4.6 × 2.5 mm (5 µm packing) Luna C18 guard column (Phenomenex, Torrance, CA, USA). The mobile phase was ACN: 20 mM phosphate buffer pH 7.0 (30:70, *v*/*v*) flowing at 1.0 mL/min. The column and auto-sampler were maintained at 25 °C. The sample volume of 20 μL was injected into the system and detected at 280 nm. Two injections per samples were analyzed by HPLC to demonstrate reproducibility of the data. Data collection and analysis were performed using OpenLab software (Agilent Technologies, Santa Clara, Wilmington, DE, USA).

#### 2.2.7. Dissolution

The dissolution of the core and coated tablets was performed using USP apparatus 2 (Model 708-DS with 850-DS autosampler, Agilent Technologies Santa Clara, CA, USA). The dissolution of the core tablets was performed in 900 mL 0.2 M phosphate buffer pH 6.8 at 50 rpm and 37 °C. Samples (1 mL) were collected at 45 min.

The dissolution of the coated tablets was performed in 500 mL 0.1 N HCl at 50 rpm and 37 °C for 2 h. The sample (1 mL) was collected at 2 h. The dissolution of the coated tablets was also performed in 900 mL 0.2 M phosphate buffer 6.8 at 50 rpm and 37 °C for 24 h and the samples were collected at 1, 2, 4, 6, 8 and 24 h interval and filtered through 70 µm filter. A 20 µL sample was injected into the HPLC system to quantitate the amount of drug dissolved. The dissolution of the tablets was performed in triplicate.

#### 2.2.8. Statistical Analysis

JMP Pro 15 (SAS, Cary, NC, USA) and UnscramblerX (version 10.1; Camo Process, Oslo, Norway) were used in the data analysis.

## 3. Results

### 3.1. Film Characterization

#### 3.1.1. Surface Morphology

**Figure 1:**
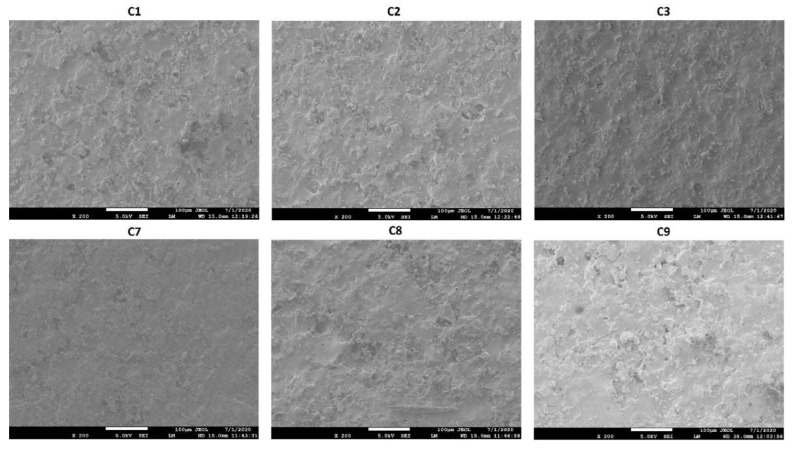
Surface morphology of tablets coated with blends of cellulose acetate butyrate (CAB) 171-15/C-A-P.

The surface morphology of the coated tablets showed rough surface with pinholes, cratering, and crevices. However, the degree of surface deformation decreased with an increase in the coating weight. The C3 formulation (coated at 10.75% weight gain) showed less deformity than C1 (coated at 5.21% weight gain). Similarly, no effects of C-A-P or CAB 171-10 polymer percentage in the coating formulation on surface morphology were observed (Figure 1).

#### 3.1.2. Near Infrared Hyperspectroscopy

**Figure 2:**
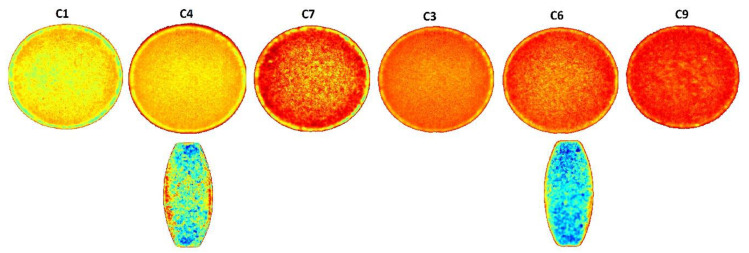
Principal component analysis (PCA) images of tablets coated with blends of CAB 171-15/C-A-P.

Near infrared hyperspectroscopy provided chemical and spatial information about the samples. Hypercube data were base line corrected and mathematically treated by standard normal variate to generate principal component analysis (PCA) images. The PCA images of the coated tablets and half-cut tablets are shown in Figure 2. The pixel color was relatively uniform in the coated tablets. Pixel colors changed from yellow to red (C1, C4, and C7) with the composition of the polymer blend in 5% coated tablets. The pixel color did not change significantly in 10% coated (C3, C6, and C9). Furthermore, coating thickness can be visualized in the coated tablets. As expected, the coating was relatively thinner in the 5% coated tablets compared to the 10% coated ones. The core tablets can also be visualized from half-cut tablets as it showed multicolored pixels indicating the multicomponent nature of the core tablets.

### 3.2. Dissolution Models by Design of Experiment Approach

The friability, hardness, disintegrationm and dissolution of the core tablets were less than 1% *w*/*w*, 6–7 kP, 4–5 min and >75%. The coated tablets did not disintegrate in 0.1 N HCl and less than 0.5% drug was released in the 0.1 N HCl dissolution medium.

**Figure 3:**
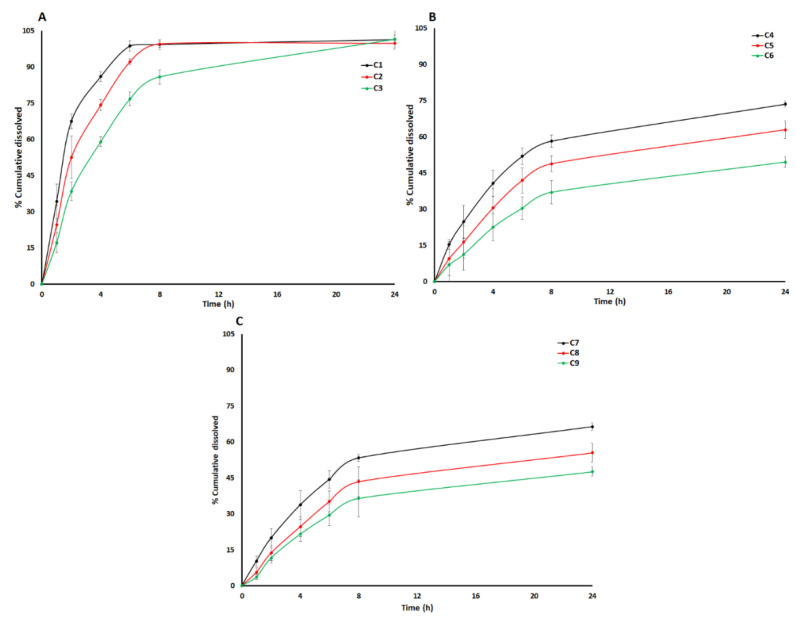
Dissolution profiles of tablet coated with CAB 171-15/C-A-P at approximately (**A**) 5%, (**B**) 7.5%, and (**C**) 10% weight gain.

The dissolution varied as a function of the coating composition and coating weight gain. The thickness of the coating on the core tablet increased with an increase in the percentage coating weight gain. The C-A-P polymer and PEG dissolved during the dissolution process leaving behind a porous film of CAB 171-15 polymer with micro-holes. The degree of porosity controls the rate of penetration of the dissolution medium and drug diffusion. A thicker film leads to a decreased permeability of the dissolution medium to penetrate the tablet and less diffusion of the dissolved drug [36]. Increasing the CAB 171-10 content of the blends resulted in decreased porosity and decreased the dissolution rate (Figure 3). The coating shell remained intact after 24 h when the coating composition contained 50% or more CAB 171-15 relative to C-A-P. This explained the shorter duration of the sustained dissolution (4–8 h) in the formulations containing low percentages of CAB 171-15 (C1–C3, 33.3%) compared to ones (C4–C9, 50–66.7%) containing a high percentage of CAB 171-15. A higher percentage of CAB 171-15 in the coating composition resulted in a more compact film that remained intact for longer time and thus sustained the drug dissolution for longer duration (C4–C9). Dissolution profiles can be divided into three distinct phases, namely initial, middle, and later (Figure 3).

The initial, middle, and later phases can be described by 1 (Y_1_), 8 (Y_2_), and 24 h (Y_3_) dissolution time points. The effect of variables on the responses Y_1_, Y_2,_ and Y_3_ can be described by the following equations:

Y_1_ = 14.6 − 5.3X_1_ − 10.2X_2_ + 1.9X_1_X_2_ (1)

Y_2_ = 62.6 − 9.1X_1_ − 26.6X_2_ − 2.5X_1_X_2_ (2)

Y_3_ = 72.8 − 7.5X_1_ − 23.3X_2_ − 5.8X_1_X_2_ (3)

**Figure 4:**
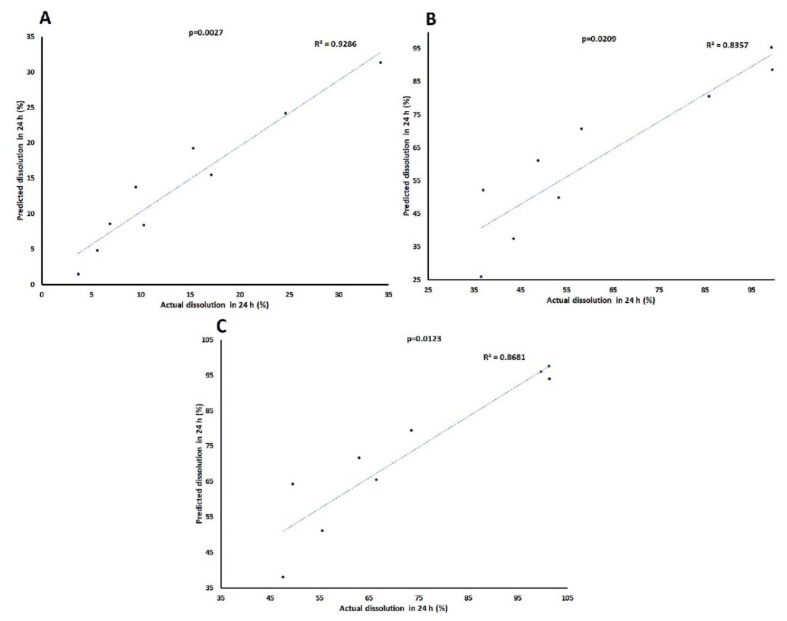
Actual and model predicted curve of (**A**) Y_1_, (**B**) Y_2_, and (**C**) Y_3_ tablets coated with CAB 171-15/C-A-P blend.

An independent variable increases the dependent variable if its sign is positive in the equation and vice versa [30]. The model showed a good correlation between actual and predicted values as indicated by the correlation coefficients of 0.964, 0.914, and 0.932 for Y_1_, Y_2_, and Y_3_, respectively (Figure 4). The model of Y_1_ can explain the greater percentage of variability in the data compared to Y_2_ and Y_3_. The models of Y_1_, Y_2,_ and Y_3_ can explain the 96.4, 83.6, and 86.8% variability in data, respectively. Moreover, error in the models was measured by residual and root mean squared errors (RMSE). The residual values varied from −4.2 to 2.9, −15.3 to 10.8, and −14.9 to 9.5 for Y_1_, Y_2,_ and Y_3_, respectively. Similarly, the RMSE value was 3.4, 13.1, and 10.2 for Y_1_, Y_2_, and Y_3_, respectively. Thus, error in Y_1_ was low compared to Y_2_ and Y_3_.

**Figure 5:**
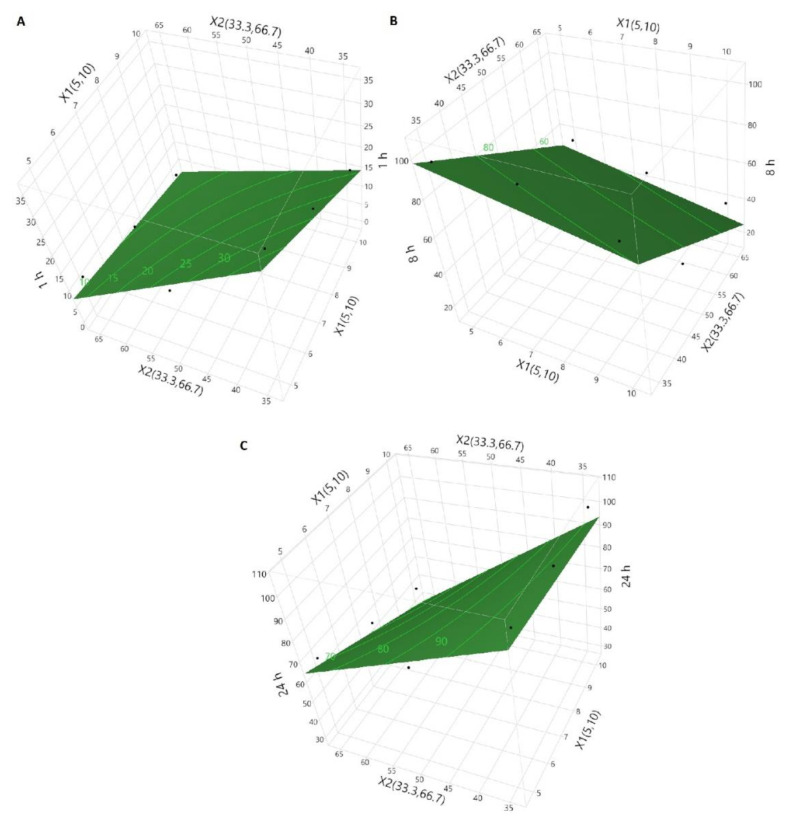
Response surface curves of (**A**) Y_1_, (**B**) Y_2_, and (**C**) Y_3_ tablets coated with CAB 171-15/C-A-P blend.

Statistically significant (*p* < 0.05) effects of X_1_ on Y_1_ and X_2_ on Y_1_, Y_2,_ and Y_3_ were observed. The independent variables had a negative influence over Y_1_, Y_2,_ and Y_3_ (Figure 5).

**Figure 6:**
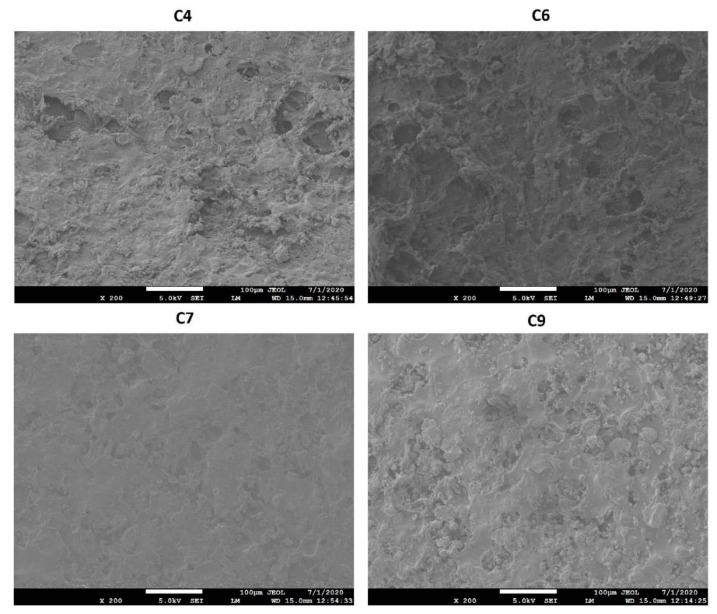
Surface morphology of shell of the tablets coated with blends of CAB 171-15/C-A-P blend after dissolution test.

The values of dissolution decreased with an increase in the coating percentage (coating weight gain). This was due to an increase in the thickness of the polymer membrane over the core tablet that controls drug diffusion from core tablet to the bulk dissolution medium. A thicker membrane means that the drug will encounter greater resistance when diffusing the membrane [36]. Furthermore, C-A-P polymer dissolved during the dissolution leaving behind a shell of CAB 171-15 polymer encasing the core tablet. This also increased the tortuosity of the path that the drug had to travel to cross the thicker membrane (Figure 6). The C1 and C3 formulations were coated with the same coating composition but coated at 5.2 and 10.8% weight gain, respectively. The values of Y_1_, Y_2_, and Y_3_ in the C1 and C3 formulations were 34.2 ± 7.1, 99.3 ± 2.0 and 101.3 ± 1.8%, and 17.1 ± 4.1, 85.8 ± 3.0, and 101.4 ± 4.2%, respectively. Furthermore, the rate and extent of dissolution can be changed with coating composition, especially the percentage of CAB 171-10 relative to C-A-P polymer. Dissolution decreased with an increase in CAB 171-10 polymer percentage from 33.3% (C1−C3) to 66.7% (C7−C9). The drug was released through the micropores formed by the solubilization of PEG and C-A-P during the dissolution.

Since the PEG concentration was constant in all the coating compositions, the micropores size and number were entirely depend upon C-A-P in the coating composition. The size and number of micropores decreased with a decrease in C-A-P percent in the coating formulation as less C-A-P was available for the micropores formation that resulted in decreased dissolution. For examples, Formulation C4 and C7 were coated at 5.4 and 4.5% coating weight gain, respectively, but the coating formulation contained 50.0% and 66.7% CAB 171-10 relative to C-A-P. Dissolution at 1, 8, and 24 h from C4 and C7 formulations was 15.3 ± 2.0, 58.2 ± 2.5 and 73.5 ± 1.2%, and 10.3 ± 2.0, 53.3 ± 1.6, and 66.4 ± 1.5%, respectively. The micropore size was small in C7 compared C4 that had a higher percentage of C-A-P (Figure 6).

Analysis of variance (ANOVA) at *p* < 0.05 was performed to determine whether the effect of independent variables on the dependent variables was real or by chance. ANOVA indicated a significant effect of the independent variables on the dependent variables (*p* < 0.05).

The models were verified by independent data not used in the model development. The coating composition and coating percentage was selected that would dissolve more than 50% drug in 24 h. The core tablet was coated at 9.8% weight gain with coating composition containing 60% CAB 171-15, and 40% C-A-P. The model predicted value of Y_1_, Y_2,_ and Y_3_ responses were 4.6, 36.8, and 48.8%, respectively. Empirical values of Y_1_, Y_2_, and Y_3_ were 1.2 ± 0.2, 30.2 ± 4.5, and 58.6 ± 5.2%, respectively. The residual was low for Y_1_ (3.4%) and Y_2_ (6.6%), and high for Y_3_ (9.8%) response.

### 3.3. Dissolution Models by Chemometric Approach

#### 3.3.1. NIR Spectra

**Figure 7:**
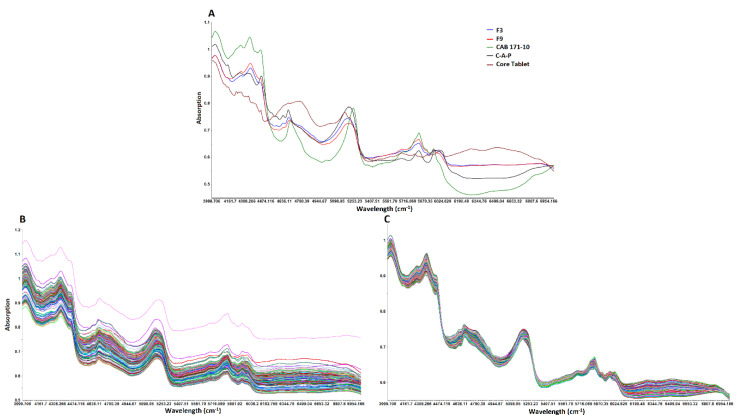
NIR spectra of (**A**) core, coating components, and coated tablets, (**B**) raw and (**C**) multiplicative scattered corrected.

NIR spectra of the coating formulation component, core, and coated tablets are shown in Figure 7A. NIR spectra of core tablet was different from polymers used in the blend. C-A-P showed major peaks at 4142, 4435, 4624, 4670, 5203, and 5820 cm^−1^. CAB 171-10 showed distinct peaks at 4331, 4427, 4690, 5241, 5816, and 5943 cm^−1^. The coated tablets showed the peaks of both polymers. The intensity and characteristics of peaks change as the composition of coating formulation changes. With an increase in CAB 171-10 polymer in the coating composition, the spectra incorporated more features of CAB 171-10 than C-A-P. It was also characterized by changes in slope and intercept of the spectra (Figure 7A). These spectral changes can be linked to the coating composition and dissolution responses to build chemometric prediction models.

#### 3.3.2. Data Pretreatment, Outlier and Number of Latent Variables

There were differences in the baselines of the spectra of the replicate samples. This was due to physical variation in the replicate samples such as refractive index, packing density, and surface morphology. These factors contribute to varying/effective sample path-length which resulted in additive, multiplicative, and wavelength dependent effects. This is manifested in the baseline shift, tilt, or curvature in the spectra. These effects can be eliminated or reduced by mathematical treatment of the data. The most commonly used methods are standard normal variate, multiplicative scattering correction, derivatives, and so forth [37,38]. The NIR spectral data were mathematically corrected by multiplicative scattering correction (MSC) to eliminate or reduce variation in data of replicate samples due base-line shift and non-linearity. The corrected data were analyzed for RMSE and R^2^. The RMSE and R^2^ values of mathematically untreated and treated data were 11.9 and 0.68, and 4.8 and 0.949, respectively. Furthermore, replicate data were overlapping each other after MSC treatment (Figure 7B,C). Additionally, outliers in the data were detected by Hotelier T_2_ at *p* < 0.05. The Hotellings T_2_ statistics limit was 8.12 and all the samples were within this limit.

**Figure 8:**
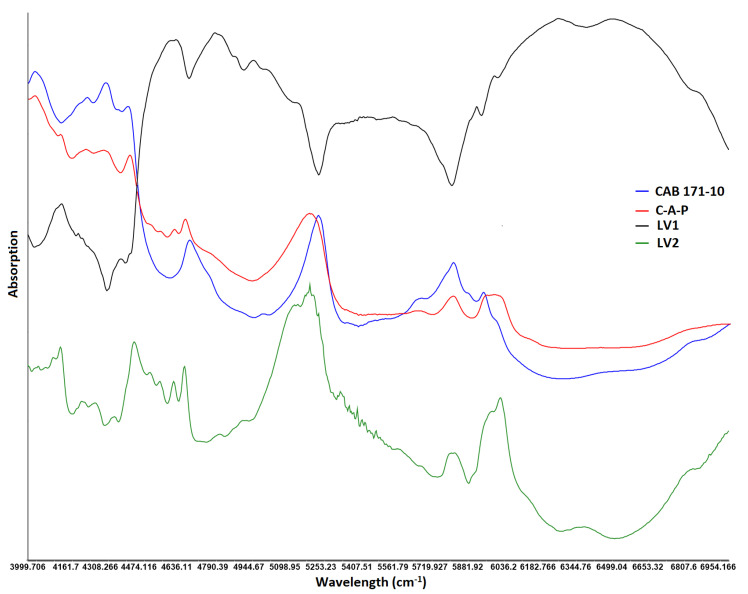
NIR spectra of latent variables and coating components.

The over and under fitting of the models were determined by number of latent variables (LV). The optimum number of LV was determined by assessing RMSE and R^2^. The value of RMSE and R^2^ were 15.9 and 0.458, 4.8 and 0.949, and 4.7 and 0.952 for first, second, and third LV, respectively. Increasing LV beyond two did not significantly change values of RMSE and R^2^. Therefore, two LV were selected for model development. Furthermore, LVs are related to physical and chemical information of the spectra. The first and second LV were compared with the individual component of the coating formulation. LV1 showed peaks of CAB 171-15, although in inverted position while LV2 showed peaks of both C-A-P and CAB 171-15 (Figure 8).

#### 3.3.3. Models Development and Validation

**Figure 9:**
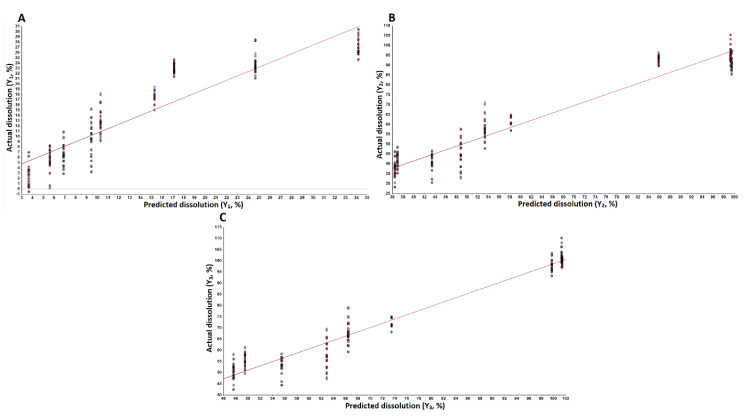
Chemometric models predicted and actual values curve of (**A**) Y_1_, (**B**) Y_2_ and (**C**) Y_3_ tablets coated with CAB 171-15/C-A-P blend.

Mathematically treated NIR data ranging from 4000 to 7000 cm^−1^ were used for the development of partial least squares regression (PLSR) models as this range showed maximum changes in the spectra (Figure 7C). The cross-validation approach was employed for PLSR model development. In the cross-validation approach, same data set was utilized to internally validate the model. The data for formulations C1–C9 was used for the model development for Y_1_, Y_2_, and Y_3_ responses. The model showed a good correlation between actual and model predicted values for the responses. The correlation coefficient was 0.916, 0.964, and 0.974 for Y_1_, Y_2_, and Y_3_, respectively (Figure 9). The error in the model was measured by residual, standard error (SEC) and RMSE (RMSEC) of calibration. The residual between actual dissolution and model predicted values ranged from −7.6 to 9.4, −14.6 to 14.8, and −13.7 to 14.2 for Y_1_, Y_2_, and Y_3_, respectively. SEC and RMSEC measure the precision and accuracy of the model. The SEC and RMSE for Y_1_, Y_2_, and Y_3_ were 3.87 and 3.88, 6.58 and 6.59, and 4.85 and 4.87, respectively. Thus, the model for Y_1_ was more accurate and precise compared to Y_2_ and Y_3_.

The statistical parameters of internally validated model should be close to the calibration for a well fitted model. Over fitting of model was indicated by lower values of R, R^2^, SEP, and RMSEP of the prediction model compared to the R, R^2^, SEC, and RMSEC of the calibration model. The statistical parameter values of the validation model were close to those of the calibration model (Table 2). Furthermore, the models were further validated by an independent sample not used in the development. In the independent sample, the core tablet was coated 9.8% weight gain with a coating composition containing 60% CAB 171-15 and 40% C-A-P. The data of independent sample was truncated and mathematically treated with MSC. The model predicted values were close to the empirical values. Additionally, error in the values was low as indicated by the residual. The experimental values of Y_1_, Y_2,_ and Y_3_ were 1.2 ± 0.2, 30.2 ± 4.5, and 58.6 ± 5.2%, respectively. The models predicted values were 5.6 ± 3.0, 38.5 ± 7.1, and 61.3 ± 6.2% for Y_1_, Y_2_ and Y_3_, respectively. The difference between actual and model predicted values were 4.4, 8.3, and 2.7 for Y_1_, Y_2_, and Y_3_ responses, respectively.

## 4. Conclusions

Predictive dissolution models of a cellulose ester blend were developed by the design of experiment and chemometric methods. CAB 171-15 and coating weight gain had a negative effect on dissolution. Rate and extent of dissolution can be modulated by CAB 171-15 proportion relative to C-A-P and coating weight gain. Increasing these two variables increased the thickness of film through which the drug diffused into the bulk dissolution medium. The model shows good correlation for Y_1_, Y_2_, and Y_3_. Models based on NIR data show a better correlation between empirical and predicted values and low error as indicated by RMSE values than the design of experiment approach. However, the predicted values of the independent samples of both models are similar. Furthermore, the dissolution model based on NIR method provide quick way of measuring the dissolution.

## Figures and Tables

**Table 1:** Independent variables and experimental matrix.

Independent Variables	Level		
	Low	Medium	High
Coating (X_1_, %)	5.0	7.5	10.0
CAB 171-15 (X_2_, %)	33.3	50.0	66.7
**Formulation**	**X_1_ (%)**	**X_2_ (%)**	**Actual Coating Gain (%)**
C1	5	33.3	5.2
C2	7.5	33.3	7.7
C3	10	33.3	10.8
C4	5	50.0	5.3
C5	7.5.	50.0	7.9
C6	10	50.0	10.3
C7	5	66.7	4.5
C8	7.5	66.7	7.2
C9	10	66.7	9.7

**Table 2:** Statistical parameters of partial least square models.

Response	Model	Sample No.	Slope	Offset	Correlation	R^2^	RMSEC (P) ^1^	SEC (P) ^2^	Bias
Y_1_	Calibration	170	0.84	2.26	0.916	0.839	3.87	3.88	2.97 × 10^−6^
	Validation	170	0.84	2.31	0.913	0.834	3.95	3.96	0.03
Y_2_	Calibration	170	0.93	4.4	0.964	0.929	6.58	6.59	7.74 × 10^−6^
	Validation	170	0.93	4.63	0.962	0.927	6.75	6.77	0.09
Y_3_	Calibration	170	0.95	3.58	0.974	0.949	4.85	4.87	0
	Validation	170	0.95	3.98	0.973	0.948	4.97	4.99	0.06

RMSEC (P) ^1^—Root mean square error of calibration or prediction. SEC (P) ^2^—Standard error of calibration or prediction.
